# Inflammation biomarkers in blood as mortality predictors in community-acquired pneumonia admitted patients: Importance of comparison with neutrophil count percentage or neutrophil-lymphocyte ratio

**DOI:** 10.1371/journal.pone.0173947

**Published:** 2017-03-16

**Authors:** Jose Curbelo, Sergio Luquero Bueno, José María Galván-Román, Mara Ortega-Gómez, Olga Rajas, Guillermo Fernández-Jiménez, Lorena Vega-Piris, Francisco Rodríguez-Salvanes, Belén Arnalich, Ana Díaz, Ramón Costa, Hortensia de la Fuente, Ángel Lancho, Carmen Suárez, Julio Ancochea, Javier Aspa

**Affiliations:** 1 Department of Internal Medicine, Hospital Universitario de La Princesa, Instituto de Investigación Sanitaria del Hospital Universitario de la Princesa, Madrid, Spain; 2 Biobank, Hospital Universitario de La Princesa, Instituto de Investigación Sanitaria del Hospital Universitario de la Princesa, Madrid, Spain; 3 Department of Immunology, Hospital Universitario de La Princesa, Instituto de Investigación Sanitaria del Hospital Universitario de la Princesa, Madrid, Spain; 4 Department of Pneumology, Hospital Universitario de La Princesa, Instituto de Investigación Sanitaria del Hospital Universitario de la Princesa, Madrid, Spain; 5 Clinical Information Unit, Hospital Universitario de La Princesa, Instituto de Investigación Sanitaria del Hospital Universitario de la Princesa, Madrid, Spain; 6 Methodology Unit, Instituto de Investigación Sanitaria del Hospital Universitario de la Princesa, Madrid, Spain; 7 Department of Pneumology, Hospital del Henares, Madrid, Spain; 8 Department of Clinical Analysis, Hospital Universitario de La Princesa, Instituto de Investigación Sanitaria del Hospital Universitario de la Princesa, Madrid, Spain; Institut national de la recherche scientifique, CANADA

## Abstract

**Introduction:**

The increase and persistence of inflammation in community-acquired pneumonia (CAP) patients can lead to higher mortality. Biomarkers capable of measuring this inadequate inflammatory response are likely candidates to be related with a bad outcome. We investigated the association between concentrations of several inflammatory markers and mortality of CAP patients.

**Material and methods:**

This was a prospective study of hospitalised CAP patients in a Spanish university hospital. Blood tests upon admittance and in the early-stage evolution (72–120 hours) were carried out, where C-reactive protein, procalcitonin, proadrenomedullin, copeptin, white blood cell, Lymphocyte Count Percentage (LCP), Neutrophil Count Percentage (NCP) and Neutrophil/Lymphocyte Ratio (NLR) were measured. The outcome variable was mortality at 30 and 90 days. Statistical analysis included logistic regression, ROC analysis and area-under-curve test.

**Results:**

154 hospitalised CAP patients were included. Patients who died during follow-up had higher levels of procalcitonin, copeptin, proadrenomedullin, lower levels of LCP, and higher of NCP and NLR. Remarkably, multivariate analysis showed a relationship between NCP and mortality, regardless of age, severity of CAP and comorbidities. AUC analysis showed that NLR and NCP at admittance and during early-stage evolution achieved a good diagnostic power. ROC test for NCP and NLR were similar to those of the novel serum biomarkers analysed.

**Conclusions:**

NLR and NCP, are promising candidate predictors of mortality for hospitalised CAP patients, and both are cheaper, easier to perform, and at least as reliable as the new serum biomarkers. Future implementation of new biomarkers would require comparison not only with classic inflammatory parameters like White Blood Cell count but also with NLR and NCP.

## Introduction

Community acquired pneumonia (CAP) is a frequent and severe infection associated with a high mortality [1]. This mortality is not only associated with the acute episode stage; there is an increase in late mortality in CAP patients, regardless of their age and level of comorbidity [2–4]. Therefore, identifying those patients with a higher risk of mortality, at diagnosis or during their early evolution, is a clinical objective to anticipate possible complications and thereby adequately programme their follow-up.

Various predictive, initial prognostic scores have been developed, such as CURB65 [5] and Pneumonia Severity Index (PSI) [6], validated on diverse populations [7,8]. However, to improve the predictive power of these scales, different inflammatory markers have been studied such as C-reactive protein (CRP) and white blood cell count (WBC). Unfortunately, their capacity to identify patients likely to die in the long and short term is not better than that of the classic scores [9,10]. New biomarkers like procalcitonin (PCT), proadrenomedullin (proADM) and copeptin have good predictive value for mortality, at least as accurate as classic clinical scores [11–15]. Nevertheless, in spite of this, none has had sufficient in-hospital usage [16].

Recently, the Neutrophil/Lymphocyte Ratio (NLR) has been reported to be related with mortality and prognosis in CAP, with a better profile than CRP [17]. Compared with WBC, NLR would reflect more precisely the balance between neutrophil and lymphocyte responses and could be better associated with the severity of the inflammatory response.

Thus, the aim of this study was to evaluate the capacity for mortality prediction in hospitalized CAP patients at 30 and 90 days of NLR, and also Neutrophil and Lymphocyte count percentages (NCP and LCP, respectively), by comparing their prognostic proficiency with that of several serum biomarkers (CRP, PCT, ProADM and Copeptin) and with current clinical scores (PSI and CURB-65).

## Material and methods

### Overall design

This was a prospective cohort study of CAP patients who were admitted between October 2013 and July 2015 in Hospital Universitario de La Princesa, a public hospital for 300,000 inhabitants in Madrid, Spain. Inclusion criteria were being over 18 years old, having a diagnosis of CAP in the emergency room and being subsequently hospitalised for this reason. All patients included signed the informed consent form. The study protocol was approved by the Ethics Committee of the Hospital Universitario de La Princesa. The diagnosis of CAP was established by the presence of lower respiratory tract infection symptoms together with the appearance of new infiltrate in chest x-ray, and the absence of an alternative diagnosis. The decision for hospitalisation was made by medical staff according to recommendations of ATS/IDSA guidelines [18]. From all patients included in the study, a blood sample was taken in the first 24 hours of admittance and then a follow-up sample was taken 72–120 hours after admittance. Treatment and hospital discharge decisions were made by medical staff; members of the study group did not influence in clinical decision making. Follow-up of the study was set at 90 days. Mortality for any reason within 30 and 90 days were considered the main outcome variable. More information about population and recorded clinical variables could be consulted in the protocol for the study, previously published [19]. All data generated during this research are openly available from Zenodo.org public repository at DOI: 10.52181/zenodo.265245.

### Laboratory procedures

Routine analytical parameters were determined with blood samples taken upon admittance and the 72–120 hour follow-up as stated in the protocol [19]. Then, concentrations of the following inflammatory markers were measured: CRP, PCT, Pro-ADM and copeptin. Also, WBC values, neutrophil and lymphocyte counts were gathered and thus, likewise, the relative numbers of neutrophils and lymphocytes with respect to total leukocytes (NCP and LCP respectively). The NLR was obtained for each patient as the ratio of neutrophils to lymphocytes.

CRP concentrations were analysed from serum turbidimetric immunoassay with latex particles coated with anti-CRP, monoclonal antibodies, using the CRPL3 kit (Roche Diagnostics) in the cobas c 701 module analyser (Roche/Hitachi).

PCT levels were analysed from serum electrochemiluminescent immunoassay using the Elecsys PCT kit (BRAHMS) in the cobas e 602 module analyser (Roche/Hitachi).

Concentrations of pro-ADM and copeptin were analysed in EDTA plasma, using inmunofluorescence MR-proADM Kryptor (BRAHMS) and Copeptin US Kryptor kit (BRAHMS), in Kryptor Compact PLUS robot (BRAHMS).

The levels of leukocytes and their classification were measured in EDTA blood samples by semiconductor laser flow cytometry determining the leukocyte differential by means of the XT-2000i automated Hematology Analyser (Sysmex).

### Statistical analysis

Descriptive analysis was carried out with frequencies and percentages for categorical variables and mean and standard deviation for quantitative variables.

Analysis of the relationship between qualitative variables was carried out using the chi-square test or Fisher's exact test, and comparison of means was performed using a t-test or their nonparametric equivalent. Association between NCP and LCP was evaluated by Spearman correlation. In order to study the biomarkers as a predictive factor, logistic regression was used, adjusting for the potentially confounding variables. Model construction was done according to the principle of parsimony (selection processes p < 0.15). All values of p < 0.05 were considered to be statistically significant. ROC analysis was carried out for the different markers. The cut-off points for NLR and proADM were analysed taking into account the existing available literature. In case of the NCP, given the absence of available literature, two cut-off points were chosen after weighing up the optimum sensitivity and specificity. The statistical analysis was carried out using the program Stata 13.0.

## Results

A total of 154 patients requiring hospitalisation due to CAP were included. The cumulative incidence of mortality at 30 days was 7.79% (IC 95%: 4.51–13.13) and at 90 days it was 12.99% (IC 95%: 8.57–19.21). The principal characteristics of the patients, with reference to mortality at 30 and 90 days, are shown in Table 1.

**Table 1:** Description of population at 30 and 90 days follow-up. Analysis of Differences Found between Biomarkers.

	Mortality at 30 days			Mortality at 90 days		
Variables*	Non survivors	Survivors	p	Non survivors	Survivors	p
	(n = 12)	(n = 142)		(n = 20)	(n = 134)	
Age	90.4 (5.1)	74.5 (16.1)	**0.000**^W^	90.5 (4.5)	73.5 (16.0)	**0.000**^W^
Male	5 (41.7)	84 (59.2)	0.239^J^	9 (45.0)	80 (59.7)	0.214^J^
Vaccination:						
* Influenzavirus*	4 (36.4)	89 (64.0)	0.104^F^	11 (57.9)	82 (62.6)	0.693^J^
* Pneumococcus*	4 (36.4)	58 (41.7)	0.728^F^	8 (42.1)	54 (41.2)	0.942^J^
Tobacco use			0.425^F^			0.140^F^
Active smoker	1 (8.3)	31 (22.3)		2 (10.0)	30 (22.9)	
Former smoker	5 (41.7)	61 (43.9)		7 (35.0)	59 (45.0)	
Never smoker	6 (50.0)	47 (33.8)		11 (55.0)	42 (32.1)	
Pack-years^+^	48.3 (33.3)	42.4 (23.6)	0.758^W^	46.3 (32.6)	42.4 (26.5)	0.831^W^
**Comorbidities:**						
Asthma	0	8 (5.7)	1.000^F^	1 (5.0)	7 (5.3)	1.000^F^
COPD	3 (25.0)	45 (32.1)	0.754^F^	5 (25.0)	43 (32.6)	0.497^J^
Obesity	1 (8.4)	27 (19.4)	0.697^F^	3 (15.0)	25 (19.1)	1.000^F^
Diabetes Mellitus	2 (16.7)	23 (16.6)	1.000^F^	8 (25.0)	17 (12.7)	0.168^F^
Hypertension	6 (50.0)	83 (59.3)	0.531^J^	13 (65.0)	76 (57.6)	0.530^J^
Dislypidemia	5 (41.7)	44 (31.4)	0.525^F^	8 (40.0)	41 (31.1)	0.425^J^
Stroke	3 (25.0)	19 (13.4)	0.381^F^	3 (15.0)	26 (19.9)	0.766^F^
Chronic Kidney Disease	1 (8.3)	21 (15.0)	1.000^F^	2 (10.0)	20 (15.2)	0.740^F^
Heart failure	2 (16.7)	27 (19.4)	1.000^F^	3 (15.0)	26 (19.9)	0.766^F^
Dementia	7 (58.3)	19 (13.6)	**0.001**^F^	13 (65.0)	13 (9.9)	**0.000**^F^
Malnutrition	4 (33.3)	13 (9.35)	**0.031**^F^	6 (30.0)	11 (8.4)	**0.012**^F^
Aspiration pneumonia	5 (41.7)	8 (5.7)	**0.001**^F^	7 (35.0)	6 (4.6)	**0.000**^F^
HIV	0	9 (6.4)	1.000^F^	0	9 (6.8)	0.607^F^
**Severity**						
Pneumonia severity index:			**0.043**^F^			**0.005**^F^
I	0	6 (4.2)		0	6 (4.5)	
II	0	24 (16.9)		0	24 (17.9)	
III	0	26 (18.3)		0	26 (19.4)	
IV	5 (41.7)	55 (38.7)		12 (60.0)	48 (35.8)	
V	7 (58.3)	31 (21.8)		8 (40.0)	30 (22.4)	
CURB65:			**0.005**^F^			**0.005**^F^
1	0	26 (18.3)		0	26 (19.4)	
2	1 (8.3)	29 (20.4)		2 (10.0)	28 (20.9)	
3	5 (41.7)	51 (35.9)		8 (40.0)	48 (35.8)	
4	3 (25.0)	34 (23.9)		7 (35.0)	30 (22.4)	
5	3 (25.0)	2 (1.4)		3 (15.0)	2 (1.5)	
**Biomarkers at admittance**						
C reactive protein (mg/dl)	18.8 (6.8)	13.4 (9.7)	**0.019**^W^	15.0 (7.5)	13.6 (9.9)	0.346^W^
Procalcitonin (ng/ml)	10.8 (21.9)	1.4 (2.8)	**0.020**^W^	7.4 (17.3)	1.4 (2.8)	**0.026**^W^
Copeptin (pmol/l)	148.7 (283.9)	19.4 (24.3)	**0.001**^W^	92.0 (210.4)	18.8 (24.6)	**0.000**^W^
Proadrenomedullin (nmol/l)	2.8 (2.0)	1.2 (0.7)	**0.000**^W^	2.3 (1.6)	1.2 (0.8)	**0.000**^W^
Platelets (x 10^3^/mm^3^)	255 (184)	237 (209)	0.791^W^	241 (202)	238 (209)	0.917^W^
WBC (x 10^3^/mm^3^)	11.9 (8.1)	11.9 (5.2)	0.560^W^	11.5 (6.7)	12.0 (5.2)	0.488^W^
NCP (%)	89.2 (4.7)	77.9 (12.4)	**0.001**^W^	85.6 (8.0)	77.8(12.6)	**0.002**^W^
LCP (%)	6.8 (3.9)	13.3 (9.6)	**0.007**^W^	7.9 (3.9)	13.5(9.8)	0.09^W^
Monocytes (%)	3.6 (1.6)	7.4 (4.4)	**0.001**^W^	5.2 (3.6)	7.4 (4.4)	**0.003**^**W**^
NLR	16.8 (9.0)	10.5 (10.2)	**0.007**^W^	14.0 (8.4)	10.5 (10.5)	**0.013**^W^
**Biomarkers in the early-stage evolution**						
C reactive protein (mg/dl)	8.3 (5.3)	4.7 (5.5)	**0.011**^W^	7.8 (5.0)	4.6 (5.5)	**0.002**^W^
Procalcitonin (ng/ml)	2.0 (2.6)	0.4 (1.0)	**0.003**^W^	1.5 (2.1)	0.4 (1.0)	**0.000**^W^
Copeptin (pmol/l)	56.3 (51.5)	15.0 (22.1)	**0.002**^W^	39.6 (41.9)	14.5 (22.1)	**0.005**^W^
Proadrenomedullin (nmol/l)	2.5 (1.0)	1.1 (0.6)	**0.000**^W^	2.1 (0.9)	1.1 (0.6)	**0.000**^W^
Platelets (x 10^3^/mm^3^)	304 (221,5)	236 (241	0.853^W^	285 (162)	277 (136)	0.726^W^
WBC (x 10^3^/mm^3^)	13.1 (4.4)	9.1 (4.1)	**0.004**^W^	12.7 (4.9)	9.0 (3.9)	**0.001**^W^
NCP (%)	85.6 (7.1)	6.4 (13.3)	**0.001**^W^	83.0 (8.7)	65.6 (13.1)	**0.000**^W^
LCP (%)	8.1 (5.8)	22.1 (12.0)	**0.001**^W^	9.3 (5.6)	22.8 (11.9)	**0.000**^**W**^
Monocytes (%)	5.6 (3.0)	8.7 (3.5)	**0.009**^W^	6.4 (3.1)	8.8 (3.6)	**0.010**^W^
NLR	17.1 (12.8)	4.6 (4.4)	**0.000**^W^	14.7 (11.9)	4.3 (3.8)	**0.000**^W^

* Values are expressed this order: absolute number and relative frequency for categorical variables; mean and standard deviation in continuous variables, W Wilcoxon test, J chi-square test, F Fisher's exact test

COPD: Chronic obstructive pulmonary disease; HIV: Human immunodeficiency virus; LCP: Lymphocyte count percentage; NCP: Neutrophil count percentage; NLR: Neutrophil-lymphocyte ratio. WBC: white blood cells.

Patients who died in the first 30 days were older than those who survived (90.4 years old vs 74.5, p = 0.000), and they showed a higher prevalence of dementia, malnutrition, and an history of bronchial aspiration. They also had CURB65 and PSI scores higher than the survivors. Similar results were also found analysing the patients at the 90-day follow-up.

### Differences found between different biomarkers

Descriptive analysis of the different inflammatory markers and their relationship with 30-day and 90-day mortality is also shown in Table 1. Additional data about of lymphocyte and neutrophil counts are shown in S1 and S2 Tables. Patients who died in the first 30 days had, in the admittance blood test, significantly higher levels on average of CRP (18.8 mg/dL vs 13.4 mg/dL), PCT (10.8 ng/ml vs 1.4 ng/ml), copeptin (148.7 pmol/l vs 19.4 pmol/l) and proADM (2.8 nmol/l vs 1.2 nmol/l), and also a higher NCP (89.2% vs 77.9%) and NLR (16.8 vs 10.5). Both lymphocyte count and LCP were lower in patients who died (0.7 · 10^3^/mm^3^ vs 1.33 10^3^/mm^3^; 6.8% vs 13.3%). In the 90-day mortality analysis similar results were obtained, with the exception of CRP which showed a difference that did not reach the significance threshold.

In the early-stage evolution blood samples (72–120 hours), significantly higher levels were found for patients who died in the first 30 days, compared with those who survived, for the following: CRP (8.3 mg/dL vs 4.7 mg/dL), PCT (2.0 ng/ml vs 0.4 ng/ml), copeptin (56.3 pmol/l vs 15.0 pmol/l), proADM (2.5 nmol/l vs 1.1 nmol/l), WBC (13.1 · 10^3^/mm^3^ vs 9.1 · 10^3^/mm^3^), neutrophil count (11.4 · 10^3^/mm^3^ vs 6.3 · 10^3^/mm^3^), NCP (85.6% vs 66.5%) and NLR (17.1 vs 4.6). By contrast, lymphocyte count was lower in deceased patients (0.9 · 10^3^/mm^3^ vs 1.8 · 10^3^/mm^3^) and also LCP (8.1% vs 22.1%). Similar results were obtained in the early-stage evolution samples for all these inflammatory markers in the 90-day mortality analysis. Consistently, CRP, PCT, copeptin, proADM, LCP, NCP and the NLR showed significant differences between survivors and non-survivors at 30 and at 90 days in both the admission and the early-stage evolution measurements. Therefore, the remainder of the study was focused on these markers.

### Relationship between selected markers and mortality

As can be seen in Table 2, in a regression analysis (after adjustment for the following confounding variables: age, gender, CURB65, COPD, dementia, malnutrition and bronchial aspiration background), high levels on admittance of CRP, proADM and NCP, and lower levels of LCP were independently and significantly associated with mortality at 30 days, and to a lesser extent at 90 days. In the early-stage evolution blood test, all markers analysed were related with mortality statistically significative or closed.

**Table 2:** Univariate and multivariate analysis of the relationship between mortality and selected markers.

	30 days follow-up				90 days follow-up			
	OR univariate	P	OR multivariate*	P	OR univariate	p	OR multivariate*	p
**Biomarkers at admittance**								
C reactive protein	1.05	0.069	1.11	**0.015**	1.01	0.561	1.07	0.077
	(1.0–1.1)		(1.0–1.2)		(1.0–1.1)		(1.0–1.2)	
Procalcitonin	1.17	0.010	1.13	0.119	1.14	0.017	1.13	0.166
	(1.0–1.3)		(1.0–1.3)		(1.0–1.3)		(1.0–1.4)	
Copeptin	1.03	0.003	1.02	0.181	1.02	0.005	1.01	0.445
	(1.0–1.1)		(1.0–1.0)		(1.0–1.1)		(1.0–1.0)	
Proadrenomedullin	2.77	0.002	2.47	**0.028**	2.45	0.002	1.84	0.072
	(1.4–5.3)		(1.0–5.4)		(1.4–4.3)		(0.9–3.6)	
Lymphocyte count percentage	0.84	0.017	0.87	0.142	0.88	0.012	0.95	0.504
	(0.7–1.0)		(0.7–1.0)		(0.8–1.0)		(0.8–1.1)	
Neutrophil count percentage	1.2	0.002	1.26	**0.026**	1.09	0.009	1.02	0.511
	(1.1–1.4)		(1.0–1.4)		(1.1–1.2)		(0.9–1.1)	
Neutrophil/ Lymphocyte ratio	1.04	0.060	1.04	0.293	1.03	0.175	1.00	0.864
	(1.0–1.1)		(1.0–1.1)		(1.0–1.1)		(0.9–1.1)	
**Biomarkers in the early-stage evolution**								
C reactive protein	1.09	0.060	1.09	0.230	1.08	0.036	1.12	0.091
	(1.0–1.2)		(0.9–1.3)		(1.0–1.2)		(1.0–1.3)	
Procalcitonin	1.59	0.010	8.77	**0.017**	1.55	0.015	8.02	**0.003**
	(1.1–2.3)		(1.5–52.3)		(1.1–2.2)		(2.03–1.8)	
Copeptin	1.03	0.009	1.04	**0.040**	1.02	0.014	1.02	0.079
	(1.0–1.1)		(1.0–1.1)		(1.0–1.1)		(1.0–1.1)	
Proadrenomedullin	4.75	0.000	3.16	**0.035**	4.56	0.000	2.48	0.051
	(2.0–11.4)		(1.1–9.2)		(2.0–10.2)		(1.0–6.2)	
Lymphocyte count percentage	0.79	0.001	0.83	**0.033**	0.81	0.000	0.83	**0.033**
	(0.7–0.9)		(0.7–1.0)		(0.7–0.9)		(0.7–1.0)	
Neutrophil count percentage	1.19	0.000	1.16	**0.016**	1.15	0.000	1.13	**0.009**
	(1.1–1.3)		(1.0–1.3)		(1.1–1.2)		(1.0–1.2)	
Neutrophil/ Lymphocyte ratio	1.19	0.000	1.18	**0.003**	1.22	0.000	1.23	**0.002**
	(1.1–1.3)		(1.1–1.3)		(1.1–1.4)		(1.1–1.4)	

* Model adjusted by age, gender, CURB65, COPD, dementia, malnutrition and bronchial aspiration background.

### ROC curves and cut-off point analysis for the selected markers

**Fig 1:**
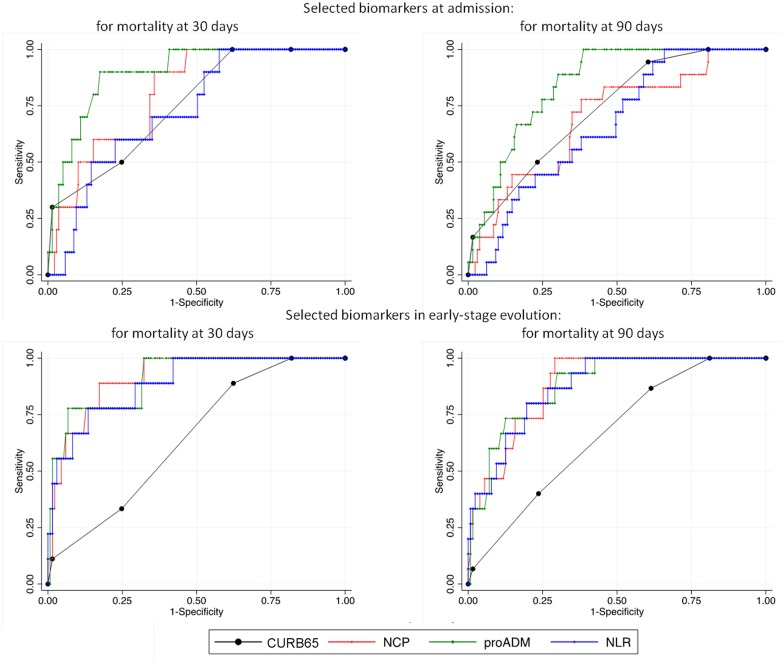
ROC Curves of CURB65 and selected biomarkers: Neutrophil Count Percentage (NCP), Proadrenomedullin (ProADM) and Neutrophil-Lymphocyte Ratio (NLR), in Blood Test at Admittance (Upper Panels) and in Early-Stage Evolution (Lower Panels), Referred to Mortality at 30 and 90 Days.

On the basis of the data shown so far, an evaluation of the predictive capacity of the different selected markers was performed by an area-under-the curve (AUC) analysis. The results are shown in Table 3 and in Fig 1. To make graphics clearer we chose the markers from each category showing the best global AUC values; proADM from the new biomarkers, and NCP and NLR from the blood parameters, since they showed a slightly better AUC values than LCP. Besides, a strong correlation was found between NCP and LCP (Spearman coefficient -0.91, p <0.001) but NCP is a marker widely used in the clinic for the analytical control of infections. CURB65 was selected from the clinical scores because is the most used in literature for comparison with new biomarkers.

**Table 3:** AUC for classic risk scales and different inflammatory markers.

	AUC (CI 95%)	AUC (CI 95%)
	for mortality at 30 days	for mortality at 90 days
Pneumonia severity index	0.76 (0.67–0.86)	0.71 (0.63–0.80)
CURB65	0.72 (0.59–0.86)	0.71 (0.61–0.82)
**On admittance blood test**		
C-reactive Protein	0.70 (0.58–0.83)	0.57 (0.44–0.69)
Procalcitonin	0.70 (0.55–0.86)	0.65 (0.53–0.78)
Copeptin	0.84 (0.73–0.96)	0.79 (0.69–0.90)
Proadrenomedullin	0.89 (0.81–0.96)	0.84 (0.77–0.91)
Lymphocyte count percentage	0.74 (0.64–0.90)	0.68 (0.58–0.79)
Neutrophil count percentage	0.83 (0.73–0.93)	0.72 (0.59–0.84)
Neutrophil-lymphocyte ratio	0.76 (0.63–0.88)	0.69 (0.58–0.79)
**Early-stage evolution blood test**		
C-reactive Protein	0.74 (0.61–0.87)	0.73 (0.63–0.83)
Procalcitonin	0.79 (0.65–0.92)	0.77 (0.67–0.87)
Copeptin	0.84 (0.71–0.97)	0.73 (0.59–0.88)
Proadrenomedullin	0.91 (0.82–0.99)	0.89 (0.81.0.96)
Lymphocyte count percentage	0.87 (0.77–0.98)	0.86 (0.78–0.94)
Neutrophil count percentage	0.90 (0.83–0.97)	0.86 (0.79–0.94)
Neutrophil-lymphocyte ratio	0.88 (0.79–0.98)	0.86 (0.79–0.94)

In order to predict mortality at 30 days, the greatest AUCs upon admission were for proADM (0.89), copeptin (0.84) and NCP (0.83); they were similar to those of the PSI and CURB65 scales. A bigger effectiveness was demonstrated by these markers in the early-stage evolution blood test, in which the AUC for NCP (0.90) was higher than that of either PSI (0.76) or CURB65 (0.72) scales, in a statistically significant manner (p = 0.035, p = 0.010 respectively), and almost the same than proADM (0.91). When these analyses were carried out for mortality at 90 days, results were similar: NCP was a better mortality marker in the early-stage evolution (AUC = 0.86) than PSI score and CURB65 scores (p = 0.002 and p = 0.005 respectively). It is remarkable that proADM did not display a significant better predictive capacity than NCP in any of the different scenarios analysed (p> 0.05).

The great predictive capacity of proADM, NCP and NLR shown in these results pointed to their possible clinical interest to predict mortality at 30 and 90 days. Therefore optimal cut-off points were determined with the corresponding calculation for sensitivity, specificity and positive and negative predictive values (Table 4), where bold characters indicate best sensitivity and specificity in each analysed situation.

**Table 4:** Cut-Off points of ProADM, NLR and NCP for mortality at 30 and 90-days.

Cut-off points	Mortality at 30 days				Mortality at 90 days			
At admittance	Sensitivity	Specificity	PPV	NPV	Sensitivity	Specificity	PPV	NPV
**ProADM**								
> 1.8	63.6	**87.1**	28.0	96.8	47.4	**87.8**	36.0	92.0
	(35.4–84.8)	**(80.5–91.6)**	(14.3–47.6)	(92.1–98.7)	(27.3–68.3)	**(81.1–92.3)**	(20.2–55.5)	(86.0–95.6)
> 1.3	90.0	64.0	16.7	98.9	**89.5**	67.2	28.3	97.8
	(62.3–98.4)	(55.8–71.5)	(9.3–28.0)	(94.0–99.8)	**(68.6–97.1)**	(58.7–74.6)	(18.5–40.8)	(92.3–99.3)
**NLR**								
> 10	63.6	65.0	12.5	95.8	52.6	65.2	17.9	90.5
	(35.4–84.3)	(56.8–72.4)	(6.2–23.6)	(89.7–98.4)	(31.7–72.7)	(56.7–72.7)	(10.0–29.8)	(83.0–94.9)
> 9	72.7	60.7	12.7	96.6	63.2	61.4	19.0	92.0
	(43.4–90.3)	(52.4–68.4)	(6.7–23.1)	(90.5–98.8)	(41.0–80.9)	(52.8–69.2)	(11.2–30.4)	(84.5–96.1)
**NCP**								
> 85%	63.7	70.5	14.3	96.1	47.4	69.7	18.4	90.2
	(35.4–84.8)	(62.0–77.0)	(7.1–26.7)	(90.3–98.5)	(23.3–68.3)	(61.4–76.9)	(10.0–31.4)	(82.9–94.6)
> 80%	**100**	47.9	13.1	100	84.2	48.5	19.1	95.5
	**(74.1–100)**	(39.8–56.1)	(7.5–21.9)	(94.6–100)	(62.4–94.5)	(40.1–56.9)	(12.1–28.7)	(87.6–98.5)
**In early-stage evolution**	Sensitivity	Specificity	PPV	NPV	Sensitivity	Specificity	PPV	NPV
**ProADM**								
> 1.8	55.6	93.5	35.7	97.0	37.5	94.0	42.9	92.5
	(26.7–81.1)	(88.1–96.5)	(16.3–61.2)	(92.5–98.9)	(18.5–61.3)	(88.4–96.9)	(21.4–67.4)	(86.7–95.9)
> 1.3	77.8	69.6	14.3	98.0	**81.3**	72.5	26.5	96.9
	(45.3–93.7)	(61.4–76.6)	(7.1–26.7)	(92.9–99.4)	**(57.0–93.4)**	(64.3–79.4)	(16.2–40.3)	(91.4–99.0)
**NLR**								
> 10	70.0	91.9	38.9	97.7	50.0	92.3	44.4	93.7
	(39.7–89.2)	(84.8–95.4)	(20.3–61.4)	(93.3–99.2)	(28.0–72.0)	(86.4–95.8)	(24.6–66.3)	(88.2–96.8)
> 9	70.0	91.2	36.8	97.6	50.0	91.5	42.1	93.7
	(39.7–89.2)	(85.2–94.9)	(19.1–59.0)	(93.3–99.2)	(28.0–72.0)	(85.5–95.2)	(23.1–63.7)	(88.1–96.8)
**NCP**								
> 85%	50.0	**95.6**	45.5	96.3	37.9	**96.1**	54.5	92.6
	(23.7–76.3)	**(90.7–98.0)**	(21.3–72.0)	(91.6–98.4)	(18.5–61.4)	**(91.3–98.3)**	(28.0–78.7)	(86.9–95.9)
> 80%	**80.0**	82.4	25.0	98.2	68.8	83.8	34.4	95.6
	**(49.0–94.3)**	(75.1–87.8)	(13.4–42.1)	(93.8–99.5)	(44.4–85.8)	(76.6–89.2)	(20.5–51.7)	(90.1–98.1)

NPV: negative predictive value; PPV: positive predictive value; proADM: proadrenomedullin; NLR: Neutrophil-lymphocyte ratio; NCP: Neutrophil count percentage. In bold characters, the best value found.

For proADM, two cut-off points were analysed, 1.8 nmol/l proposed by Christ-Crain *et al*. [20], with a specificity of 87.1%, and 1.3 nmol/l suggested by Huang *et al*. [12], which showed a sensitivity of 90.0%, both for mortality at 30 days. For NLR the cut-off point of 10 (put forward by Jager *et al*. [17]) presented a sensitivity of 63.6% and a specificity of 65% for the admission analysis and 30-day mortality, with a high negative predictive value (NPV) of 95.8. Similarly, the data for sensitivity and specificity with a cut-off point of 9 for the NLR are shown.

For NCP, no studies were found in this regard, therefore a conventional cut-off point of 85% was proposed. It showed a sensitivity for mortality at 30 days of 63.7% and a NPV of 96.1%.

In the early-stage evolution the 85% cut-off point showed a sensitivity of 50%, and specificity of 95.6%; similar values were obtained for mortality at 90 days. A cut-off point of 80% was also assayed with higher sensitivity but lower specificity.

## Discussion

In recent years there has been a race to find new biomarkers related with inflammation in CAP. Copeptin, a vasopressin surrogate molecule for PCT, peptide precursor of calcitonin rising in bacterial infection, or proADM, an adrenal gland molecule, are better mortality predictive markers than long-standing inflammatory parameters such as CRP or total WBC at admission [20–22]. Nevertheless none of these studies have done comparison with leukocyte parameters such as NLR, NCP or LCP.

In the present study we tried to evaluate some immune cell quantitative parameters, potentially associated with inflammatory response, as putative markers associated with risk of mortality, and compare them with these novel biomarkers and classic clinical scores. We found that WBC derived parameters NCP, LCP and NLR could have a prognostic power as optimal as proADM, copeptin or PCT in evaluating medium-term mortality risk (30-day and 90-day follow-up) after hospitalisation due to CAP. A summary of the main white blood cell parameters is shown in the S1 and S2 Tables. In addition, these relative white blood cell parameters of NCP, LCP and NLR, in the early-stage evolution (at 72–120 hours) also have a good predictive power.

NLR has already been described as a predictive marker for mortality in different cancers [23,24], and in different infection models such as urinary sepsis or acute endocarditis [25,26]. Jager *et al*. showed that NLR at admittance worked as a better prognostic marker in CAP than CRP and WBC, although they did not compare them with novel biomarkers or with clinical scores [17].

In the present study of patients admitted for CAP, NCP, LCP and NLR were associated with a substantial increase in the risk of mortality, and this increase was consistently maintained after adjustment for age, severity and other comorbidities.

According to our results both elevated neutrophil percentage and reduced lymphocyte percentage could be good prognostic markers in pneumonia, with results well above those obtained for absolute numbers (data shown in S1 and S2 Tables). These parameters could reflect an altered inflammatory response, with sustained neutrophil activity, associated with bad evolution of pneumonia. Our hypothesis is that this sustained inflammatory response, rather than the acute infectious process, determines mid-term bad evolution. Although any of the white cell parameters studied could be used as mortality risk parameter, we focussed on NLR because it takes into account the combined contribution of neutrophils and lymphocytes and has been well documented in the literature as a prognostic marker. We also focussed on NCP because it is a marker widely used in the clinic for the analytical control of infections, has a strong correlation with LCP, and is easier to obtain than NLR.

The good association of NLR and NCP with the risk of mortality suggests that they could be considered as candidate predictive markers in CAP. We analysed their predictive capacity performing a sensitivity/specificity analysis by ROC curve tests where AUC values are indicative of the predictive quality. We compared our analysis for NLR and NCP with other biomarkers in several recently published reports. Table 5 summarises results of the most powerful mortality predictor of each study, rated according to AUC values. Throughout these studies proADM seems to be the best of these new biomarkers, although its superiority over the classic clinical scales, PSI or CURB65 is not clear [12,27,28]. Besides, there were not comparisons between proADM and NCP or NLR in any of these studies.

**Table 5:** Studies of prognostic biomarkers and clinical scores in community acquired pneumonia.

Authors	Year	Outcome variable	Prognosis biomarkers proposed	AUC	AUC PSI	AUC CURB65
Christ-Crain *et al*. [20]	2006	Treatment failure, even death	proADM	0.73	0.73	
Huang *et al*. [12]	2009	30-day overall mortality	proADM	0.76	0.83	0.80
Kruger *et al*. [14]	2010	28-day overall mortality	proADM	0.85		0.73 *
Albrich *et al*. [27]	2011	30-day overall mortality in respiratory infection	proADM	0.79		0.73
Kolditz *et al*. [13]	2012	30-day mortality or ICU admission	Copeptin	0.81	0.75	0.57 *
Jager et *al*. [17]	2012	In-hospital mortality	NLR	0.70		
Courtais *et al*. [29]	2013	30-day overall mortality	proADM	0.80	0.71	
España *et al*. [28]	2015	30-day pneumonia-related complications	proADM	0.84	0.83	0.79
Bello *et al*. [30]	2015	30-day overall mortality	proADM	0.82	0.85	0.82
Curbelo *et al*. (current study)	2016	30-day overall mortality, in admission blood test	proADM	0.89	0.76	0.72
			NCP	0.83		
			NLR	0.76		

AUC: area-under-curve; proADM: proadrenomedullin; NLR: Neutrophil/Lymphocyte Ratio; NCP: Neutrophil Count Percentage; PSI: Pneumonia Severity Index; ICU: Intensive Care Unit;

* CRB65 (urea not included)

Our AUC for NLR and NCP were not inferior to AUC of proADM (0.76 and 0.83 Vs 0.89), and significantly better than other biomarkers (see Table 3). AUC of proADM for mortality at 30 days was similar to AUC in other studies (see Table 5). A cut-off point analysis is the best way to make a comparison of markers closer to clinical practice. Regarding proADM, the best cut-off is not well established yet. Therefore, two points already analysed in the literature were tested for mortality at 30 days: 1.8 nmol/l [20] with a sensitivity of 63.0% and a good specificity of 87.1% in our study, and 1.3 nmol/l [12], which showed a very good sensitivity of 90.0% and a specificity of 64.0% in our study. For NCP we tested the 80% point and the 85% point, with more acceptable values of sensitivity and specificity.

Our results also showed a better prognosis ability of immune parameters in the early-stage evolution, compared to parameters at admission. To date, few studies have included a series of blood tests in evolution of CAP. Zhydkov *et al*. [22] carried out a follow-up analysis for CAP and studied 30-day mortality. They studied PCT, CRP and the WBC at days 1, 3, 5 and 7. Their values in the final 7-day blood tests were related to mortality, at least as well or even better than the same parameters in the blood tests at admittance. Unfortunately for the comparison with our study, neither the NLR, nor the NCP were included.

In our study, data analysis of the second blood test was also very useful. For instance, a NCP higher than 80% in early stage evolution would be a marker of increased risk of death at 30 days, with an OR of 7.6 (IC 95%: 1.1–52.4) after adjustment for gender, age, severity and comorbidities. Similar results were obtained for NLR greater than 10 (OR 29.7 IC 95%: 3.4–260.2). Besides, NCP and NLR values in early-stage evolution were better mortality predictors than PSI or CURB65. The association of these parameters at admission with the risk of mortality was substantially lower. Our explanation is that NCP and NLR could reflect the systemic inflammatory state. Prolonged elevated levels of these parameters would imply a prolonged severe and uncontrolled immunological response, then resulting in the non-resolution of the systemic inflammatory process. Persistence of elevated levels of NLR and NCP could be a key factor for the worsening of patients comorbidities and therefore, for a bad outcome. These data reinforce the idea of monitoring not only the beginning of an inflammatory process but the early-stage evolution, to better predict short-term and long-term mortality.

Notwithstanding the interesting results of the study, it has several limitations. The sample could be considered to be rather small, although it was large enough to show clear statistically significant differences, and for NLR our data are also consistent with the study of Jager *et al*. [17]. Despite this limitation, given the accessibility and cost effectiveness of NLR and NCP in clinical practice, our data strongly support further examination of their predictive capacity with a solid validation cohort.

Another aspect already mentioned, is that the profile of our patients is fragile, elderly and with a high degree of comorbidity, which could affect the external validity of the study. The study was carried out in in-patients for CAP, which fulfilled criteria for admission. Because of this, the sample was not representative of the whole spectrum of CAP. It excluded patients who probably had less severe illness. Comparison between classic prognostic scales (CURB65 and PSI) and immune markers could be unbalanced, because clinical scales were overestimated: the condition of hospitalisation as inclusion criterion would select more severe patients and therefore increase the average score in both scales. On the other hand, inclusion of CAP patients that required hospitalisation permitted a close follow-up control with an early-stage evolution analysis, which in turn allowed the disclosure of some interesting results.

Another possible weakness of the study is the variability of the day of blood acquisition for the early-stage evolution test. A wide time range was chosen (from 72 to 120 hours), in order to accommodate it within the variability of daily hospital activity. Probably, the homogeneity of data was reduced, but it better fitted into usual clinical practice.

Finally, an important strength of our results is the small cost of NCP and NLR that are readily accessible in standard blood count tests. Given the high cost of daily use of new biomarkers, it would be worth to carry out a comparison of new candidate marker with these easily-measured parameters, searching for better performance before their implementation in standard clinical practice.

In conclusion, we show here that, unlike most of the predictive markers used in clinical practice, basic blood count could provide parameters with the potential of high predictive capacity for mortality in CAP patients, that are easy to handle and cost effective. Therefore, NLR and NCP deserve a thorough examination of their predictive capacity in bigger cohorts of patients. Once this capacity is firmly established, they could be considered as markers of choice unless putative candidates prove a better predictive power.

## Supporting information

S1 Table Comparative summary of lymphocyte and neutrophil parameters between survivors and non-survivors at 30-day follow-up, including values of univariate OR, multivariate OR and AUC from the statistical analyses. (DOC)

S2 Table Comparative summary of lymphocytic and neutrophilic parameters between survivors and non-survivors at 90-day follow-up, including values of univariate OR, multivariate OR and AUC from the statistical analyses. (DOC)

## Abbreviations

AUC: Area under the curve
CAP: Community-acquired pneumonia
COPD: Chronic Obstructive Pulmonary Disease
CRP: C-reactive protein
CURB65: Confusion—Urea—Respiratory rate—Blood pressure—65-years-old
LCP: Lymphocyte count percentage
NCP: Neutrophil count percentage
NLR: Neutrophil/lymphocyte ratio
NPV: Negative predictive value
OR: Odds ratio
PCT: Procalcitonin
proADM: Proadrenomedullin
PSI: Pneumonia Severity Index
PPV: Positive predictive value
ROC: Receiver operator curve
RR: Relative risk
WBC: White blood cells
