# Correction: Inflammation biomarkers in blood as mortality predictors in community-acquired pneumonia admitted patients: Importance of comparison with neutrophil count percentage or neutrophil-lymphocyte ratio

**DOI:** 10.1371/journal.pone.0212915

**Published:** 2019-02-22

**Authors:** Jose Curbelo, Sergio Luquero Bueno, José María Galván-Román, Mara Ortega-Gómez, Olga Rajas, Guillermo Fernández-Jiménez, Lorena Vega-Piris, Francisco Rodríguez-Salvanes, Belén Arnalich, Ana Díaz, Ramón Costa, Hortensia de la Fuente, Ángel Lancho, Carmen Suárez, Julio Ancochea, Javier Aspa

In the Funding section, one of the grant numbers from the funder Instituto de Salud Carlos III (ES)—European Regional Development Fund is listed incorrectly. The correct grant numbers are: PI 12/01142 and PI 15/01311.
