# Supplementary material for: Inflammation biomarkers in blood as mortality predictors in community-acquired pneumonia admitted patients: Importance of comparison with neutrophil count percentage or neutrophil-lymphocyte ratio
Source: PLoS One. 2017 Mar 16;12(3):e0173947. doi: 10.1371/journal.pone.0173947 (PMC5354424; doi:10.1371/journal.pone.0173947)
Supplement: S1 Table — (DOC) [file pone.0173947.s001.doc]

**S1 Table:** Comparative summary of lymphocyte and neutrophil parameters between survivors and non-survivors at **30-day follow-up,** including values of univariate OR, multivariate OR and AUC from the statistical analyses.

| Variables^*^ |  | Non survivors |  | Survivors | p |  | OR | p | OR ^&^ | p |  |  |
| --- | --- | --- | --- | --- | --- | --- | --- | --- | --- | --- | --- | --- |
|  |  |  |  |  |  |  | Univariate (CI 95%) |  | Multivariate (CI 95%) |  |  | AUC |
|  |  | (n = 12) |  | (n = 142) |  |  |  |  |  |  |  | (CI 95%) |
| **On admittance blood test** | | | | | | | | | | | | |
| Lymphocytes count |  | 0.72 (0.41) |  | 1.33 (0.73) | **0.002^w^** |  | 0.11 | **0.005** | 0.09 | 0.051 |  | 0.77 |
| ( x 10^3^/mm^3^) |  |  |  |  |  |  | (0.02-0.51) |  | (0.01-1.01) |  |  | ( 0.64-0.90) |
| LCP (%) |  | 6.84 (3.88) |  | 13.31 (9.63) | **0.007^w^** |  | 0.84 | **0.017** | 0.87 | 0.142 |  | 0.74 |
|  |  |  |  |  |  |  | (0.73- 0.97) |  | (0.72-1.05) |  |  | (0.61-0.87) |
| Neutrophils count |  | 10.71 (7.47) |  | 9.60 (4.98) | **0.083^w^** |  | 1.03 | 0.478 | 0.99 | 0.939 |  | 0.51 |
| ( x 10^3^/mm^3^) |  |  |  |  |  |  | (0.93 - 1.15) |  | (0.86- 1.13) |  |  | (0.32-0.72) |
| NCP (%) |  | 89.15 (4.70) |  | 77.93 (12.41) | **<0.001^w^** |  | 1.20 | **0.002** | 1.25 | **0.026** |  | 0.83 |
|  |  |  |  |  |  |  | (1.07 - 1.35) |  | (1.02 - 1.53) |  |  | (0.72 - 0.93) |
| Neutrophil/Lymphocyte Ratio (NLR) |  | 16.8 (9.0) |  | 10.5 (10.2) | **0.007^W^** |  | 1.04 | 0.06 | 1.02 | 0.459 |  | 0.76 |
|  |  |  |  |  |  |  | (1.0 -1.1) |  | (1.0-1.1) |  |  | (0.63 - 0.88) |
| **Early-stage evolution blood test** | | | | | | | | | | | | |
| Lymphocytes count |  | 0.89 (0.42) |  | 1.80 (0.97) | **<0.001^w^** |  | 0.07 | **0.003** | 0.17 | 0.100 |  | 0.83 |
| ( x 10^3^/mm^3^) |  |  |  |  |  |  | (0.01-0.41) |  | (0.02-1.40) |  |  | (0.71-0.95) |
| LCP (%) |  | 8.15 (5.82) |  | 22.12 (11.96) | **<0.001^w^** |  | 0.79 | **0.001** | 0.83 | **0.033** |  | 0.87 |
|  |  |  |  |  |  |  | (0.69-0.91) |  | (0.70-0.98) |  |  | (0.77-0.98) |
| Neutrophils count |  | 11.41(4.45) |  | 6.35 (3.83) | **<0.001^w^** |  | 1.24 | **0.001** | 1.32 | **0.013** |  | 0.84 |
| ( x 10^3^/mm^3^) |  |  |  |  |  |  | 1.09 - 1.40) |  | (1.06 - 1.63) |  |  | (0.73 - 0.93) |
| NCP (%) |  | 85.63 (7.12) |  | 66.45 (13.30) | **<0.001^w^** |  | 1.19 | **<0.001** | 1.15 | **0.016** |  | 0.90 |
|  |  |  |  |  |  |  | (1.08- 1.30) |  | (1.02 - 1.30) |  |  | (0.82 - 0.97) |
| Neutrophil/Lymphocyte Ratio (NLR) |  | 17.1 (12.8) |  | 4.6 (4.4) | **<0.001^w^** |  | 1.19 | **<0.001** | 1.17 | **0.005** |  | 0.88 |
|  |  |  |  |  |  |  | (1.1-1.3) |  | (1.1- 1.3) |  |  | (0.79-0.98) |

LCP: Lymphocyte Count Percentage; NCP: Neutrophil Count Percentage * Values are expressed this order: mean and standard deviation in continuous variables, W Wilcoxon test & Model adjusted by age, gender, CURB65, COPD, dementia, malnutrition and bronchial aspiration background.
