# Supplementary material for: Inflammation biomarkers in blood as mortality predictors in community-acquired pneumonia admitted patients: Importance of comparison with neutrophil count percentage or neutrophil-lymphocyte ratio
Source: PLoS One. 2017 Mar 16;12(3):e0173947. doi: 10.1371/journal.pone.0173947 (PMC5354424; doi:10.1371/journal.pone.0173947)
Supplement: S2 Table — (DOC) [file pone.0173947.s002.doc]

**S2 Table:** Comparative summary of lymphocytic and neutrophilic parameters between survivors and non-survivorsat **90-day follow-up**, including values of univariate OR, multivariate OR and AUC from the statistical analyses.

| Variables^*^ | |  | | Non survivors | |  | Survivors | | p | |  | | OR | | p | | OR ^&^ | | p | |  | |  |
| --- | --- | --- | --- | --- | --- | --- | --- | --- | --- | --- | --- | --- | --- | --- | --- | --- | --- | --- | --- | --- | --- | --- | --- |
|  |  |  |  |  |  |  |  |  |  |  |  |  | Univariate | |  |  | Multivariate | |  |  |  |  | AUC |
|  |  |  |  | (n = 12) | |  | (n = 142) | |  |  |  |  | (CI 95%) | |  |  | (CI 95%) | |  |  |  |  | (CI 95%) |
| **On admittance blood test** | | | | | | | | | | | | | | | | | | | | | | | |
| Lymphocyte count |  | | 0.81 (0.37) | |  | 1.35 (0.75) | | **0.001^w^** | |  | | 0.18 | | **0.002** | | 0.27 | | 0.146 | |  | | 0.73 | |
| ( x 10^3^/mm^3^) |  |  |  |  |  |  |  |  |  |  | | (0.06-0.53) | |  |  | (0.05-1.58) | |  |  |  |  | (0.63-0.83) | |
| LCP (%) |  | | 7.95(3.89) | |  | 13.53 (9.84) | | **0.009^w^** | |  | | 0.88 | | **0.012** | | 0.95 | | 0.504 | |  | | 0.68 | |
|  |  |  |  |  |  |  |  |  |  |  |  | (0.80-0.97) | |  |  | (0.83-1.10) | |  |  |  |  | (0.58-0.79) | |
| Neutrophil count |  | | 10.03 (6.25) | |  | 9.64 (5.04) | | 0.930^w^ | |  | | 1.01 | | 0.749 | | 0.95 | | 0.458 | |  | | 0.51 | |
| ( x 10^3^/mm^3^) |  |  |  |  |  |  |  |  |  |  |  | (0.93 - 1.10) | |  |  | (0.82-1.09) | |  |  |  |  | (0.36-0.66) | |
| NCP (%) |  | | 85.59 (7.97) | |  | 77.79 (12.59) | | **0.002^w^** | |  | | 1.09 | | **0.009** | | 1.02 | | 0.511 | |  | | 0.71 | |
|  |  |  |  |  |  |  |  |  |  |  |  | (1.02 - 1.16) | |  |  | 0.94-1.12) | |  |  |  |  | (0.59-0.83) | |
| Neutrophil/Lymphocyte Ratio (NLR) |  | | 14.0 (8.4) | |  | 10.5 (10.5) | | **0.013^W^** | |  | | 1.02 | | 0.175 | | 1.00 | | 0.631 | |  | | 0.69 | |
|  |  |  |  |  |  |  |  |  |  |  |  | (1.0 - 1.1) | |  |  | (0.9-1.1) | |  |  |  |  | (0.58-0.79) | |
| **Early-stage evolution blood test** | | | | | | | | | | | | | | | | | | | | | | | |
| Lymphocytes count |  | | 0.97 (0.43) | |  | 1.84 (0.98) | | **<0.001^w^** | |  | | 0.10 | | **<0.001** | | 0.17 | | **0.044** | |  | | 0.81 | |
| ( x 10^3^/mm^3^) |  |  |  |  |  |  |  |  |  |  |  | (0.03- 0.35) | |  |  | (0.03-0.95) | |  |  |  |  | (0.72-0.91) | |
| LCP (%) |  | | 9.29 (5.63) | |  | 22.81 (11.91) | | **<0.001^w^** | |  | | 0.81 | | **<0.001** | | 0.82 | | **0.009** | |  | | 0.86 | |
|  |  |  |  |  |  |  |  |  |  |  |  | (0.74 -0.90) | |  |  | (0.71-0.95) | |  |  |  |  | (0.78-0.94) | |
| Neutrophils count |  | | 10.78 (4.91) | |  | 6.14 (3.61) | | **<0.001^w^** | |  | | 1.24 | | **<0.001** | | 1.36 | | **0.003** | |  | | 0.80 | |
| ( x 10^3^/mm^3^) |  |  |  |  |  |  |  |  |  |  |  | (1.11 - 1.39) | |  |  | (1.10-1.68) | |  |  |  |  | (0.70 - 0.89) | |
| NCP (%) |  | | 82.97 (8.74) | |  | 65.65 (13.09) | | **<0.001^w^** | |  | | 1.15 | | **<0.001** | | 1,12 | | **0.009** | |  | | 0.86 | |
|  |  |  |  |  |  |  |  |  |  |  |  | (1.08 - 1.23) | |  |  | (1.03-1.23) | |  |  |  |  | (0.78-0.93) | |
| Neutrophil/Lymphocyte Ratio (NLR) |  | | 14.7 (11.9) | |  | 4.3 (3.8) | | **<0.001^w^** | |  | | 1.22 | | **<0.001** | | 1.17 | | **0.005** | |  | | 0.86 | |
|  |  |  |  |  |  |  |  |  |  |  |  | (1.1-1.4) | |  |  | (1.1-1.3) | |  |  |  |  | (0.79-0.94) | |

LCP: Lymphocyte Count Percentage; NCP: Neutrophil Count Percentage * Values are expressed this order: mean and standard deviation in continuous variables, W Wilcoxon test & Model adjusted by age, gender, CURB65, COPD, dementia, malnutrition and bronchial aspiration background.
